# Lessons Learned With Laparoscopic Management of Complicated Grades of Acute Appendicitis

**DOI:** 10.14740/jocmr1837w

**Published:** 2014-05-22

**Authors:** Carlos Augusto Gomes, Cleber Soares Junior, Evandro de Freitas Campos Costa, Paula de Assis Pereira Alves, Carolina Vieira de Faria, Igor Vitoi Cangussu, Luisa Pires Costa, Camila Couto Gomes, Felipe Couto Gomes

**Affiliations:** a Surgery Department, Hospital Universitario (HU) Terezinha de Jesus da Faculdade de Ciencias Medicas e da Saude de Juiz de Fora (SUPREMA), Brasil; Hospital Universitario (HU) Universidade Federal de Juiz de Fora (UFJF), Brasil; b Anestesiology Unit, Hospital Terezinha de Jesus da Faculdade de Ciencias Medicas e da Saude de Juiz de Fora (SUPREMA), Brasil; c Surgical Unit, Hospital Terezinha de Jesus da Faculdade de Ciencias Medicas e da Saude de Juiz de Fora (SUPREMA), Brasil; d Internal Medicine Departament, Hospital Universitario (HU), Universidade Federal de Juiz de Fora (UFJF), Brasil; e Morphology Unit, Faculdade de Ciencias Medicas e da Saude de Juiz de Fora (SUPREMA), Brasil

**Keywords:** Appendicitis, Appendectomy, Laparoscopy, Treatment

## Abstract

**Background:**

Laparoscopy has not been consolidated as the approach of first choice in the management of complicated appendicitis. Methodological flaws and absence of disease stratification criteria have been implicated in that less evidence. The objective is to study the safe and effectiveness of laparoscopy in the management of complicated appendicitis according to laparoscopic grading system.

**Method:**

From January 2008 to January 2011, 154 consecutive patients who underwent a laparoscopic appendectomy for complicated appendicitis were evaluated in the prospective way. The patient’s age ranged from 12 to 75 years old (31.7 ± 13.3) and 58.3% were male. Complicated appendicitis refers to gangrenous and/or perforated appendix and were graded as 3A (segmental necrosis), 3B (base necrosis), 4A (abscess), 4B (regional peritonitis) and 5 (diffuse peritonitis). The outcomes including operative time, infection complication, operative complications and conversion rate were chosen to evaluate the procedure.

**Results:**

The grade 3A was the most frequent with 50 (32.4%) patients. The mean operative time was 69.4 ± 26.3 minutes. The grade 4A showed the highest mean operative time (80.1 ± 26.7 minutes). The wound and intra-abdominal infection rates were 2.6 and 4.6%, respectively. The base necrosis was the most important factor associated with the conversion (5.2%). The grades 4A and 5 were associated with greater possibility of intra-abdominal collection. There were no operative complications.

**Conclusion:**

The laparoscopic management of all complicated grades of acute appendicitis is safe and effective and should be the procedure of first choice. The laparoscopic grading system allows us to assess patients in the same disease stage.

## Introduction

Appendicitis is the most common cause of acute surgical abdomen, and despite advances in early diagnosis, it still shows non-negligible morbidity (10%) and mortality (1-5%) rates [1]. Treatment via laparotomy or laparoscopy is suitable and can be used interchangeably (level III, grade A) [2]. However, laparoscopic images allowed, with minimal incision, the safe exploration of the abdominal cavity and treatment of the different grades of the disease, but with different levels of clinical evidence [3].

Laparoscopy is considered a safe and effective approach for the treatment of uncomplicated acute appendicitis and can replace the laparotomy (level I, grade A) [2]. Regarding the complicated grades, randomized controlled trials, which compare both approaches, have not been identified yet, so controversies remain [4-6]. It is clear, however, based on observational studies, that laparoscopy is on its way to becoming the procedure of choice to manage these forms of the disease (level III, grade C) [2, 7].

In fact, the generic designation of complicated appendicitis or peritonitis is no longer appropriate and can lead to inaccurate interpretations when comparing the two approaches of management of complicated grades of acute appendicitis. It diverges from the intention of evaluating patients with the same clinical stage of the disease. It incurs in inference errors due to insufficient and/or heterogeneous samples. Therefore, it must be emphasized that research now requires greater methodological rigor, especially in relation to sample size, inclusion criteria, stratification of complicated forms and culture proof of surgical site infection [8].

To that end, the purpose of this research is to evaluate the safety and effectiveness of laparoscopy in the management of complicated acute appendicitis according to laparoscopic grading system of the disease.

## Method

From January 2008 to January 2011, 154 consecutive patients who underwent a laparoscopic appendectomy for complicated acute appendicitis were evaluated in the prospective way. The study project was approved by the Ethics and Research Commission at the Monte Sinai Hospital in Juiz de Fora, Minas Gerais, Brazil, and all patients signed up an informed consent term. Three senior surgeons who are titled by the Brazilian Society of Laparoscopic Surgery participated in it. The patient’s age ranged from 12 to 75 years old (31.7 ± 13.3) and 58.3% were male.

Complicated appendicitis refers to gangrenous and/or perforated appendix, which may lead to abscess formation and degrees of peritonitis and were graded as 3A (segmental necrosis), 3B (base necrosis), 4A (abscess), 4B (regional peritonitis) and 5 (diffuse peritonitis) according previous author’s research [9].

All patients were operated under general anesthesia and received 2 g amoxacillin-clavulanate intravenously in the perioperative period. The patients with complicated disease were then treated with double antibiotic coverage of metronidazole (1.5 g/day) and ceftriaxone (2 g/day, patients who are allergic to penicillin received ciprofloxacin 400 mg/twice daily) until white blood cell count reached normal limits and temperature was lower than 38 ^°^C. The recommended mean time of antibiotic coverage ranged from 5 to 10 days according to grade and clinical course.

The operation was performed by three ports and they were located in the umbilicus to introduce a 30^°^ Karl Storz^®^ (23006 BA) optic, in the suprapubic midline (5 mm), and in the left lower quadrant (12 mm). Then, the patient was positioned in the Trendelenburg with a mild left tilt, to facilitate the exposure of the right lower quadrant. The appendiceal stump closure was performed by applying two T 400 metal endoclips (Ethicon Endo-Surgery^®^), in a healthy tissue next to the cecum wall (distance of less than 3.0 mm) and other one around the appendix base. After the appendix section, the extraverted appendiceal mucosa was endocoagulated. Laparoscopic knot, laparoscopic endo-suture and video-assisted laparotomy were the alternatives used in difficult cases. The abdominal cavity was judiciously irrigated with warm saline solution and suctioned dry under direct visualization. Then, the appendixes were removed from the abdominal cavity in a retrieval bag and sent for histological study to confirm the diagnosis of complicated appendicitis.

Operative time that lasts from skin incision to skin suture was measured in terms of minutes. Surgical site infection was defined by clinical signs of edema, redness around the wound, or purulent discharge until the 30th postoperative day. The diagnosis of intra-abdominal infections was suspected by clinical signs and demonstrated by ecography and computed tomography. Operative complications were defined as bleeding, iatrogenic injury, post-operative small bowel obstruction and enteric leak.

The mean operative time, surgical site infection, operative complication and conversion rate were the outcomes chosen to verify the safe and effectiveness of laparoscopic appendectomy in the management of different grades of complicated appendicitis. The Statistical Package for Social Sciences (SPSS) version 13.0 for Windows was used as a database to compile all information. A descriptive statistical exploratory process was used for variable crossing. We used a 95% confidence interval, with a significance of P < 0.05.

## Results

### Frequency of complicated grades of acute appendicitis according to the laparoscopic grading system (n = 154)

Of the 154 patients operated in this series for complicated acute appendicitis, the grade 3A was the most frequent with 50 (32.4%) patients, and grade 3B was the least frequent with 14 (9.1%). We also observed that grade 4A showed considerable frequency with 43 (27.9%) patients (Table 1).

**Table 1:** Patients Underwent a Laparoscopic Appendectomy for Complicated Acute Appendicitis According to the Laparoscopic Grading System (n = 154)

Grade	N	%
Grade 3A^*^	50	32.4
Grade 3B	14	9.1
Grade 4A	43	27.9
Grade 4B	26	16.8
Grade 5	21	13.8
Total	154	100

^*^Most frequent complicated grade of acute appendicitis.

### Mean operating time spent during laparoscopic appendectomy for complicated grades of acute appendicitis compared with four other similar studies (n = 154)

The mean operative time spent during laparoscopic appendectomy for complicated grades of acute appendicitis was 69.4 ± 26.3 min. Patients with abscess presented the highest operative time (80.1 ± 26.7 min). This time was then compared with four other similar studies and represents the shortest time between them (Table 2).

**Table 2:** Mean Operating Time Spent During Laparoscopic Appendectomy for Complicated Grades of Acute Appendicitis From Four Similar Studies (n = 154)

Study or subgroup	Mean^*^	SD^#^	n^§^
Katsuno et al (2009) [17]	116.7	45	141
Lin et al (2006) [18]	96.1	43.1	99
So et al (2002) [19]	73	25	85
Khalili et al (1999) [20]	86	29	122
µGomes et al (2012)	69.4	26.3	154

^*^Mean operating time in minutes. ^#^Standard deviation. ^§^Sample of each series. ^µ^Author’s series.

### Frequency of wound and intra-abdominal infection according to the laparoscopic grading system of acute appendicitis (n = 154)

A surgical site infection was diagnosed in 11 (7.2%) patients who underwent a laparoscopic appendectomy due to complicated grades of acute appendicitis. It was diagnosed as wound infection in four (2.6%) patients and as intra-abdominal in seven (4.6%) patients. The grades 4A and 5 were associated with greater possibility of intra-abdominal infection in three (1.9%) and two (1.3%) patients respectively (Table 3).

**Table 3:** Wound and Intra-Abdominal Infection in Patients Who Underwent a Laparoscopic Appendectomy According to Laparoscopic Grading System (n = 154)

Surgical site infection	Laparoscopic grading system					Total	
	Grade 3A	Grade 3B	Grade 4A	Grade 4B	Grade 5	Event^#^	%
Wound							
Yes	0	2	0	1	1	4	2.6
No	50	12	43	25	120	150	97.4
Total	50	14	43	26	212	154	100
Intra-abdominal							
Yes	2	0	3	0	2	7	4.6
No	48	14	40	26	19	147	95.4
Total	50	14	43	26	21	154	100

^#^Event (surgical site infection by each grade).

### Conversion rate and operative complications in accordance to laparoscopic grading system of acute appendicitis (n = 154)

The conversion to laparotomy occurred in eight (5.2%) patients in this series and the majority of them belonged to grade 3B. Therefore, the presence of base necrosis represented the most important factor associated with failure of the laparoscopic procedure. On the other hand, there were no operative complications (Table 4).

**Table 4:** Conversion Rate and Operative Complications in Patients Who Underwent a Laparoscopic Appendectomy in Accordance to Laparoscopic Grading System (n = 154)

	Laparoscopic grading system					Total	
	Grade 3A	Grade 3B	Grade 4A	Grade 4B	Grade 5	Event^#^	%
Laparotomy							
Yes	0	5	2	0	1	8	5.2
No	50	9	41	26	20	142	94.8
Operative# complication							
Yes	0	0	0	0	0	0	0
N (grade)	50	14	43	26	21	154	100

Bleeding, iatrogenic injury, post-operative obstruction and enteric leak. ^#^Conversion and complication in accordance to specific grade.

## Discussion

The lack of prospective randomized studies comparing, grade by grade, laparoscopy and laparotomy in the management of complicated appendicitis is a gap in the literature. Katkhouda et al [10] emphasized that wound and intra-abdominal infection were the most important outcomes and these were traditionally used in the studies comparing both approaches. At the same time, they estimated that in order to assess the intra-abdominal infection rate, a sample of 2514 patients would be required to achieve statistical significance. Chung et al [11] found that, based on a true difference of 1% and each rate being less than 5%, 4,200 patients would need to be randomized to achieve a power of 80%. They concluded then that these figures make the research an impractical task.

Thus, the study purpose is to stratify and analyze the results of management of complicated appendicitis according to the laparoscopic grading system (Table 2). This alternative, despite not being tested in a randomized clinical trial, may contribute to sample size reduction and enable the research more feasible. In addition, it allowed investigating patients in the same clinical stage of disease to reduce the possibility of bias in the results by sample heterogeneity.

Although it has not been the object of this study, a recent meta-analysis by Markides et al [12] found no difference in operative time between the laparoscopy and laparotomy approaches. In this series, the mean operative time of 69.4 ± 26.3 min, was the lowest when compared with the four other studies [13-16], but is similar to that reported by So et al [15], in which all patients were operated by trained surgeons (Table 2). The patients classified in grade 4A had longer mean operative time (80.1 ± 26.7 min), which may explain the presence of more advanced cecum-appendiceal inflammatory processes and difficulty surgery (Table 3).

Wound infections and intra-abdominal abscess were used to validate the safe and effectiveness of laparoscopy in the management of complicated acute appendicitis in this series also. A point that should be highlighted is that the diagnosis of surgical site infection would more accurately require positive organism culture confirmation rather than just a clinical diagnosis, as used in this series and in others one [10]. In addition, we agree that much of postoperative abscess actually represents serous collection in the postoperative discharge, particularly when copious amount of irrigation fluid used. Moreover, the histological exam of the all removed appendix showed the presence of necrosis or perforation. A recent meta-analysis compiled 11 studies and has shown that from the 2,175 operated patients with complicated acute appendicitis, 92 (4.2%) had wound infection [13]. Therefore, the frequency of 2.6%, observed in this series, was low and is in agreement with the literature (Table 4). Additionally, despite the study limitations, the frequency of wound and intra-abdominal infection diagnosed in different grades of complicated acute appendicitis is first demonstrated in the literature.

The frequency of intra-abdominal infection in the same study was 5.9% (1,059 patients/63 events) [12]. Katkhouda et al [17] have reduced its frequency from 2.4% to 0.4%, with the implementation of a laparoscopic surgery service and some simple per-operative care such as exposure of the appendicular base; concern with fragments, gaps and the appendicolith; inspection, irrigation and aspiration of the bottom of the peritoneal cavity; and the use of endobags [18]. Adherence to these operative basic principles is the utmost importance for reducing intra-abdominal infection rates. The frequency of 4.6% was low. Of the seven patients with intra-abdominal collection, five of them were treated exclusively with antibiotics and the other two by percutaneous drainage. The criterion used to manage clinically was the presence of intra-abdominal collection less than 4 cm and no septic state. No patient required reoperation, and all of them had uneventful recovery (Table 3).

The need of laparotomy in the management of complicated appendicitis is variable in its frequency and can reach 10 to 39.7% [19, 20]. Among the assigned factors are adhesions, localized perforation, diffuse peritonitis, necrosis of the base of the appendix, retrocecal position, bleeding and inability to identify the organs, appendicular tumor and iatrogenic injury [3]. In this series, laparotomy was necessary in eight (5.2%) patients. Five out eight patients belonged to grade 3B. Therefore, the grade 3B represented the most important factor associated with failure of the laparoscopic procedure and conversion rate in this research. The use of mechanical stapler can circumvent the problem and reduce the conversion rate; however, because of its cost, it was not used (Table 4).

Despite research limitations, the stratification process of the disease calls attention to a better control of methodological flaws observed in previous studies, enables reduction in the patient sample size, introduces specific grades of complicated appendicitis and creates homogeneous groups of patients, when both procedures (laparotomy and laparoscopy) were compared. It presents new data found by evaluating each grade of complicated acute appendicitis. In conclusion, the laparoscopic management of all complicated grades of acute appendicitis is safe and effective and should be the procedure of first choice. The laparoscopic grading system allows us to assess patients in the same clinical stage of the disease.
